# Race, Ideology, and the Tea Party: A Longitudinal Study

**DOI:** 10.1371/journal.pone.0067110

**Published:** 2013-06-25

**Authors:** Eric D. Knowles, Brian S. Lowery, Elizabeth P. Shulman, Rebecca L. Schaumberg

**Affiliations:** 1 Department of Psychology, New York University, New York, New York, United States of America; 2 Graduate School of Business, Stanford University, Stanford, California, United States of America; 3 Positive Psychology Center, University of Pennsylvania, Philadelphia, Pennsylvania, United States of America; 4 Department of Psychology, Temple University, Philadelphia, Pennsylvania, United States of America; Hungarian Academy of Sciences, Hungary

## Abstract

The Tea Party movement, which rose to prominence in the United States after the election of President Barack Obama, provides an ideal context in which to examine the roles of racial concerns and ideology in politics. A three-wave longitudinal study tracked changes in White Americans’ self-identification with the Tea Party, racial concerns (prejudice and racial identification), and ideologies (libertarianism and social conservatism) over nine months. Latent Growth Modeling (LGM) was used to evaluate potential causal relationships between Tea Party identification and these factors. Across time points, racial prejudice was indirectly associated with movement identification through Whites’ assertions of national decline. Although initial levels of White identity did not predict change in Tea Party identification, initial levels of Tea Party identification predicted increases in White identity over the study period. Across the three assessments, support for the Tea Party fell among libertarians, but rose among social conservatives. Results are discussed in terms of legitimation theories of prejudice, the “racializing” power of political judgments, and the ideological dynamics of the Tea Party.

## Introduction

To an extent not seen in years, the country is engaged in a debate about race and politics. From claims that racial prejudice fuels opposition to President Obama and his policies [1] to assertions that Obama’s election signaled the end of racism in the U.S. [2], [3], Americans are grappling with an old question: What roles do race, racial identity, and racial prejudice play in our politics? Data now suggest that, despite hopes to the contrary, race and politics remain intertwined. Indeed, prejudice against Black Americans significantly predicted voting patterns in the 2008 general election [4]–[6] and continues to shape individuals’ policy attitudes [6], [7].

Perhaps no recent development evokes issues of race and politics more than the rise of the nearly all-White “Tea Party” movement [8]. Some commentators (e.g., [9]) have argued that the Tea Party is an *outgroup-focused* movement–an angry reaction against Blacks, Latinos, and other racial-ethnic minorities seen to threaten “traditional” American values. Others (e.g., [10]) contend that the movement is an *ingroup-focused* expression of White racial identity and anxiety in an increasingly diverse populace. For their part, Tea Party supporters insist that their movement is *principled*–rooted not in racial concerns but in ideological commitments concerning individual responsibility and the proper scope of government (e.g., [11]).

The present study was designed with two purposes in mind. First, we wanted to shed empirical light on claims that the Tea Party is–at least in part–a racial movement. In so doing, we sought to illuminate more general processes that might connect White Americans’ racial concerns and political choices. In approaching this question, we drew a distinction between two such processes: one in which racial thinking drives individuals’ politics (“politics as racialized”) and another in which politics drive individuals’ thoughts concerning race (“politics as racializing”). These processes need not be mutually exclusive; nevertheless, distinguishing them promises to add important nuance to psychological analyses of race and politics.

Our second goal was to probe the ideological origins of popular support for the Tea Party movement. A number of researchers (e.g., [12]) have argued that conservative political attitudes, while frequently *mistaken* as racial, most often reflect race-neutral ideological principles. In light of this perspective, it was important to examine the role of conservative ideology in the Tea Party movement alongside that of racial identity and prejudice. In doing this, we also sought to address a question regarding which form of conservatism–economic or social–fuels popular support for the movement.

To accomplish these goals, we conducted a three-wave longitudinal study tracking the co-development of White individuals’ racial concerns, ideologies, and identification with the Tea Party. Before describing the study, however, we first review important theoretical frameworks for understanding the interplay of racial concerns, ideology, and political judgment.

### Politics as Racializ*ed*

Most political-psychological examinations of the relationship between race and dominant-group politics fit under the “politics as racialized” rubric. This tradition can be roughly divided into two camps based on *whose* race is seen to affect Whites’ politics–racial-ethnic minorities’ (the “outgroup-focused approach”) or Whites’ (the “ingroup-focused approach”).

#### The outgroup-focused approach

An important body of work in social and political psychology posits a link between White Americans’ political judgments and their prejudices toward non-Whites. Symbolic and modern racism theories (e.g., [13], [14]), for example, emphasize the role of negative affect and racial resentment in shaping Whites’ political attitudes and behavior [13], [15].

Consistent with the outgroup-focused approach, anti-Black prejudice has been shown to predict opposition to redistributive racial policies (e.g., affirmative action) [16], support for “get tough” criminal justice initiatives [17], and a preference for conservative political candidates [18], [19]. Most recently, anti-Black prejudice was found to predict voting patterns in the 2008 general election [4]–[6], [20]. By affecting perceptions of minority politicians, racial prejudice even shapes attitudes toward nonracial policies, such as President Obama’s health care reform plan [6], [7].

#### The ingroup-focused approach

Another tradition of research links Whites’ political judgments, not to individuals’ feelings about racial outgroups, but rather to their thoughts and feelings about their own racial group. Prominent examples of this perspective are realistic group conflict [21], group position [22], [23], and certain social identity approaches [24], all of which suggest that concern for the interests or social position of the racial ingroup is an important cause of Whites’ political attitudes and behavior.

Whereas objective, fiscal self-interest has proven a weak determinant of dominant-group members’ attitudes toward public policies [25], [26], evidence suggests that self- and group-interest, broadly construed, are important factors in political judgment. For instance, support for gender-based affirmative action is sensitive to males’ and females’ feelings of collective relative deprivation [27], and individuals often judge self-interest through the prism of what is best for the group as a whole [28]. Moreover, people frequently express a desire to advantage their own group while simultaneously adopting an apathetic or even benevolent stance toward outgroups [24], [29]–[34]. In light of such findings, some theorists have argued that racial bias in the post-Civil Rights era is more likely to take the form of pro-White than anti-Black attitudes [35].

### Politics as Racializing

Perspectives that link race and politics usually assume a one-way causal relationship in which racial attitudes shape political judgments [36]. This assumption neglects the possibility that the opposite causal pathway–from political judgment to racial concerns–functions to align individuals’ political and racial thinking. We argue that political judgments (even initially-principled ones) can influence racial attitudes and identity through a process of *political racialization*. Political decisions that lead to affiliation or identification with a particular movement inevitably shape the ideas and discourses to which individuals are exposed [37]. Selective exposure to a movement’s preferred information sources and political cues can, in turn, influence the organization and content of individuals’ beliefs and attitudes [38]–[40]–including, we propose, their levels of racial prejudice and feelings of shared fate with the racial ingroup. The process of political racialization, if borne out, would imply that a person’s political judgments can be both “principled” and “racial,” originating in nonracial ideologies but nonetheless systematically altering his or her racial views.

Recent research provides evidence for such a racialization process. Sidanius and colleagues [41] examined the effects of membership in racially homogeneous fraternities and sororities–described by the authors as “ethnic enclaves”–on White college students’ intergroup attitudes. The researchers found that racial concerns (specifically, White ingroup identification) predicted entry into the Greek system. However, membership in the Greek system also increased racial identity and anti-minority affect among Whites after controlling for the students’ prior attitudes. These findings suggest that, regardless of *why* students joined fraternities and sororities, participation in those racially homogeneous organizations changed members’ racial views. In similar fashion, racially charged Tea Party rhetoric (e.g., depictions of President Obama as a noncitizen or witchdoctor) may systematically influence the racial attitudes and identity of those who choose–even for nonracial, ideological reasons–to identify with the movement.

### The Role of Ideology

Ideological commitments figure heavily in people’s politics. In particular, the liberal–conservative dimension has proven one of the most robust predictors of political attitudes and behavior [42], [43]. One school of thought, in fact, argues that racialized-politics research systematically underestimates the role of conservative principles in shaping people’s political choices [12]. According to the “principled conservatism” approach, high scores on commonly used measures of racism often reflect, not prejudice, but rather opposition to policies and views that run afoul of conservative tenets (e.g., individualism; [44]). Hence, correlations between prejudice scores and political attitudes do not by themselves demonstrate that those attitudes are racially motivated. In their critique, principled-conservatism theorists offer an important rejoinder to both the outgroup- and ingroup-focused perspectives on race and politics.

To the extent that the Tea Party is a conservative movement, what kind of conservatism does it reflect? By most accounts, the Tea Party first arose as a libertarian reaction to perceived government encroachment into the economic lives of citizens [45], [46]. Nonetheless, ongoing popular support for the movement may owe as much to social conservatism as to libertarianism. The Republican Party’s attempts to harness the Tea Party for electoral advantage [47], [48] may have increased the movement’s real or apparent alignment with culturally conservative causes, thus increasing its appeal to social conservatives. The present research attempts to evaluate this possibility.

### The Current Study

The Tea Party is an ideal phenomenon in which to examine the role of racial concerns and ideology in contemporary American politics. In its call for smaller government, rejection of wealth redistribution, and anti-elitist rhetoric, the Tea Party is just the latest incarnation of conservative populism in the United States [45]. The Tea Party, however, may be a uniquely important exemplar, with some arguing that the movement’s appeal and political influence surpass that of any previous American populist movement [45], [49]. At the same time, the Tea Party’s racial homogeneity and staunch opposition to the nation’s first Black president, as well as its noted use of racially charged rhetoric at public rallies [50], suggests potential links between individuals’ racial concerns and support for the movement.

The present longitudinal study seeks a better understanding of popular support for the Tea Party by drawing on the outgroup-focused, ingroup-focused, and ideological perspectives outlined above. In particular, we aim to discover whether the Tea Party is “racialized” or “racializing”–that is, whether racial concerns drive identification with the Tea Party or Tea Party identification shapes individuals’ racial concerns. To address this question, we analyze the co-development of White participants’ racial attitudes, ingroup identity, and identification with Tea Party over a period of nine months using latent growth modeling (LGM) [51]. This powerful correlational technique is an ideal approach for interrogating the causes of change in a construct (such as identification with the Tea Party movement) because, unlike linear regression, it enables one to simultaneously estimate the effect of the initial level of a given variable on change over time in another variable, and vice versa. Furthermore, LGM enables the examination of such effects with variation due to measurement error removed.

## Methods

### Ethics Information

The present research was conducted under the approval of the Institutional Review Board (IRB) at the University of California, Irvine (the first author’s former affiliation). After reading an IRB-approved study information page, respondents were instructed to proceed to the online survey only if they consented to participate.

### Participants

A sample of 327 self-described “White/European Americans” was recruited from a database, maintained by the Stanford Graduate School of Business, of individuals interested in completing online studies (114 males, 210 females, 3 sex not reported; aged from 20 to 71, *M* = 38.0, *SD* = 11.3 years). The survey was described as an online “study of policy views.” Three waves of data were collected: Time 1 in July 2010, Time 2 in October 2010, and Time 3 in April–May 2011. For each assessment, an email with a link to the project website was sent to participants just before the assessment was to begin. Participants were told that there “are no right or wrong answers; we are only interested in your personal perspective.” Measures (described below) were administered in the following fixed order: the Anti-Black scale, the Perceived Racial Common Fate items, the Social Dominance Orientation (SDO) scale, the Libertarian-Totalitarianism scale, the social conservatism item, the national decline items, the Tea Party support items, and the demographic questions.

An invitation to participate in the follow-up waves was conditioned on completion of the Time 1 survey. Participants were emailed a link to the study website and told that, while many of the questions had been asked previously, that “this is by design” and to “answer all the questions according to what you currently think.” Of the participants initially recruited, 250 (76.4%) completed all three assessments, and 316 (96.6%) completed at least one of the follow-up assessments. The final dataset included participants for whom some longitudinal data were available (*N* = 316). As compensation, participants received a $5 online gift certificate upon completing the Time 1 survey, a $10 gift certificate upon completing the Time 2 survey, and $15 gift certificate upon completing the Time 3 survey.

### Primary Measures

#### Ideological principles

Because small-government, libertarian principles are the stated basis of the Tea Party movement, we assessed individuals’ support for this ideology using Mehrabian’s [52] 20-item Libertarian-Totalitarianism scale (see Appendix S1). Participants made their responses on a 5-point Likert scale (1 =  *strongly disagree*, 3 =  *neutral/no opinion*, 5 =  *strongly agree*). The measure exhibited good internal reliability at Time 1 (*α* = .93), Time 2 (*α* = .93), and Time 3 (*α* = .93), with higher scores reflecting stronger endorsement of libertarian ideology.

Some commentators have argued that the Tea Party movement attracts social conservatives as well as economically conservative libertarians [53]. This view regards the Tea Party as combining small-government ideology with traditional social and cultural values. To evaluate the extent to which Tea Party identification reflects or drives social conservatism, participants were asked to rate their level of social conservatism using a single item: “When it comes to *social and cultural issues*, how would you describe your political views?” Participants made their responses on a 5-point scale (1 =  *very socially liberal*, 2 =  *somewhat socially liberal*, 3 =  *socially moderate*, 4 =  *somewhat socially conservative*, 5 =  *very socially conservative*).

#### Racial prejudice

To measure outgroup prejudice, we administered a 5-item subset of Katz and Hass’s [54] Anti-Black Scale (see Appendix S1). Participants made their responses on a 5-point Likert scale (1 =  *strongly disagree*, 3 =  *neutral/no opinion*, 5 =  *strongly agree*). The measure exhibited adequate internal reliability at Time 1 (*α* = .74), Time 2 (*α* = .76), and Time 3 (*α* = .77).

### Racial Identification

Ingroup identification was measured using a 4-item subset of Lowery, Knowles, and Unzueta’s [55] Perceived Racial Common Fate scale (see Appendix S1). This scale measures the degree to which individuals (in this case, Whites) see their fortunes as linked to that of the racial ingroup. Participants made their responses on a 5-point Likert scale (1 =  *strongly disagree*, 3 =  *neutral/no opinion*, 5 =  *strongly agree*). The measure exhibited adequate internal reliability at Time 1 (*α* = .76), Time 2 (*α* = .83), and Time 3 (*α* = .77).

The perception of common fate constitutes an important dimension of social identification [56], and is positively related to perceived ingroup entitativity (i.e., the degree to which a collection of individuals is regarded as a coherent, unitary, and distinct group) as well as other identity dimensions [57]. We reasoned that common fate might be an especially relevant facet of racial identity in the political domain; to the extent that ingroup considerations affect social perceptions and political judgment, they likely do so by rendering the group’s material prospects relevant to the self [58].

#### Tea party identification

Participants’ identification with the Tea Party was measured using the following two items: “I consider myself a supporter of the Tea Party movement” and “I would consider supporting the Tea Party by attending a local meeting or rally.” Participants made their responses to the support item on a 5-point Likert scale (1 =  *strongly disagree*, 3 =  *neutral/no opinion*, 5 =  *strongly agree*). The participation item had the same choice options, with the addition of a sixth option: 6 =  *I already have*. Only 13 participants reported having gone to a rally or meeting at any point in the study; therefore, we did not attempt to analyze attendance as a separate dependent variable. Instead, we reasoned that having attended a meeting or rally represents the highest level of willingness to participate in the Tea Party, and thus retained 6 as the maximum score possible on the item. The support and participation willingness items formed a reliable scale at Time 1 (*α* = .91), Time 2 (*α* = .92), and Time 3 (*α* = .92). For the analyses, scores on these two items were transformed such that both ranged from 0 to 4, and the mean was calculated for each time point. If the Tea Party support item is dichotomized around the midpoint to create supporter and nonsupporter categories, we find that 25% of our participants were Tea Party supporters. This is compared to 18% in a representative sample collected by the New York Times in early April 2010 [8].

### Secondary Measures

We included measures of additional constructs that previous research shows are powerful predictors of White Americans’ political attitudes and behaviors. While not our central focus, we controlled for these variables in our analyses, thus helping to clarify the roles of our primary constructs in the Tea Party movement.

#### Preference for intergroup hierarchy

Participants completed a six-item subset of Pratto and colleagues’ [59] Social Dominance Orientation (SDO) scale. SDO is conceptualized as one’s degree of general anti-egalitarian sentiment–that is, tolerance of and desire for intergroup hierarchy–and does not fit neatly into the ingroup-focused, outgroup-focused, or ideological approaches described above. However, SDO powerfully predicts a range of beliefs and attitudes whose effect is to buttress intergroup hierarchies [44], [60]–[62]. Participants made their responses on a 5-point Likert scale (1 =  *strongly disagree*, 3 =  *neutral/no opinion*, 5 =  *strongly agree*). The measure exhibited adequate internal reliability at Time 1 (*α* = .81), Time 2 (*α* = .84), and Time 3 (*α* = .85).

#### National decline

We administered two items tapping a sense of national decline–that the country, having diverged from its founding values, is becoming less and less truly “American.” This construct may reflect a sense of estrangement from the political establishment and perceived social trends [63]–[65]–a major motif in Tea Party circles [8]–[10]. The items were: “Compared to the America I grew up in, sometimes I barely recognize what this country is becoming” and “In this country, there is a ‘real America’ distinct from those who don’t share the same values.” Participants made their responses on a 5-point Likert scale (1 =  *strongly disagree*, 3 =  *neutral/no opinion*, 5 =  *strongly agree*). The items formed a reliable scale at Time 1 (*α* = .72), Time 2 (*α* = .76), and Time 3 (*α* = .73).

#### Demographics

Participants were asked to complete demographic items potentially related to political preferences: sex, years of age, and educational attainment (1 =  *less than 6 years of school*, 2 =  *some high school*, 3 =  *completed high school*, 4 =  *some college*, 5 =  *technical education*, 6 =  *college degree*, 7 =  *some graduate school*, 8 =  *a graduate degree*).

## Results

### Correlations among Constructs at Time 1

To obtain a preliminary look at relationships between Tea Party identification, racial concerns, and ideology, we examined bivariate correlations between the relevant variables at Time 1 (Table 1). Identification with the Tea Party was positively associated with anti-Black prejudice, libertarian ideology, social conservatism, Social Dominance Orientation (SDO), and national decline; Tea Party identification was negatively associated with racial identity. We also observed numerous strong correlations between these various constructs, highlighting the need to disentangle potential causal relationships using more probative analytical techniques.

**Table 1:** Means and Standard Deviations of, and Associations Between, Variables Assessed at Time 1.

	M	SD	1	2	3	4	5	6
1. Tea party identification	1.42	1.22						
2. Racial prejudice	2.91	0.75	.20***					
3. Racial identity	2.68	0.81	−.10+	.05				
4. Libertarianism	3.51	0.78	.52***	.14*	−.27***			
5. Social conservatism	2.78	1.23	.49***	.17**	−.10^+^	.54***		
6. Social Dominance Orientation	2.23	0.81	.42***	.39***	.08	.32***	.31***	
7. National decline	3.17	1.06	.58***	.23***	.04	.46***	.46***	.39***

*Note.* ^+^ *p*<.10. *p<.05. **p<.01. ***p<.001.

### Change in Tea Party Identification Over Time

As an initial step in our latent growth model (LGM) analysis, two models describing change over time in Tea Party identification (TPID) were estimated using the Mplus 5.2 software package [66]. First, a latent-intercept-only model was specified, in essence asserting that there was no true change in TPID across the three assessments (i.e., that any observed change is due to measurement error). Second, this “no growth” model was compared to one in which change over time in TPID was specified as having both a latent intercept and a latent slope (“linear growth”), allowing for the possibility of true (linear) change over time in TPID. Comparison of the chi-square fit statistics indicated that the linear growth model was superior to the no-growth model [Δ*χ* ^2^(3) = 27.14, p<.001]. Moreover, the estimated mean of the latent slope term was significantly less than zero, suggesting that, on average, participants declined over time in TPID (B = -.10, SE = .02, p<.001).

### Correlates of Change in Tea Party Identification

#### Building the structural equation model

**Figure 1:**
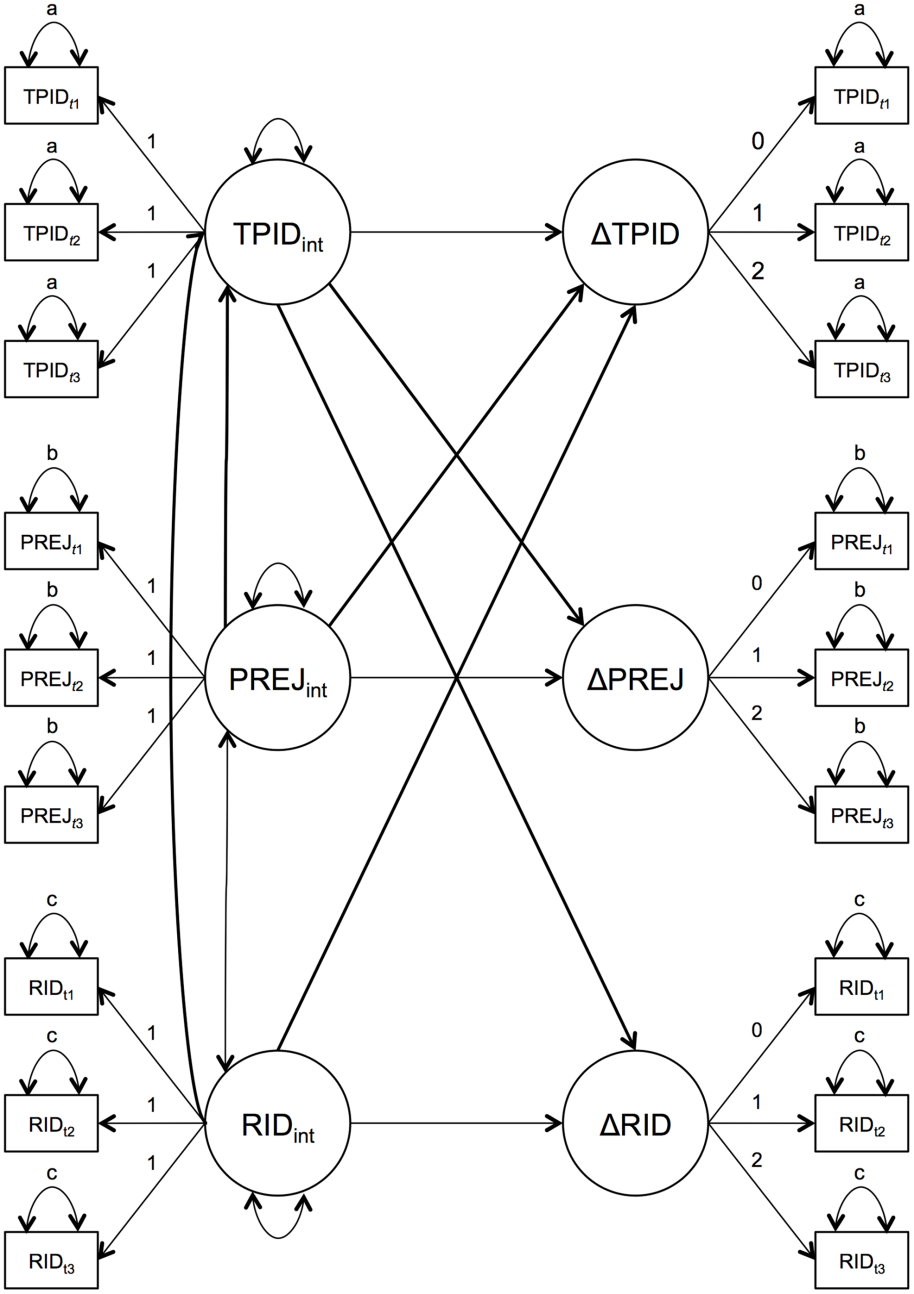
Simplified illustration of the final model. This simplified version of the final model describes the model parameters; for purposes of clarity, only two variables–racial prejudice (PREJ) and racial identity (RID)–are included to represent the six political variables modeled alongside Tea Party identification (TPID). Subscripts t1, t2, and t3 signify the three time points. Double-headed arrows represent covariance or variance parameters. Single-headed arrows represent regression paths. Variances marked with the same letter were constrained to equality. Bolded arrows highlight the paths that test the study hypotheses. All parameters estimated in the model are represented except for the regression of each intercept term on each control variable (age, sex, and education). The variances of the latent slope terms for TPID, PREJ, and RID were fixed at zero, whereas those for the other political variables (not shown) were estimated.

In order to investigate correlates of both the initial level of, and change in, TPID, a structural equation model was estimated specifying linear LGMs for each of the variables theoretically related to TPID (which we refer to collectively as the “political variables”)–outgroup prejudice, ingroup identification, SDO, libertarian ideology, social conservatism, and national decline–in addition to the linear growth model for TPID. Based on observed estimates, the variance parameters for the slopes of TPID, outgroup prejudice, and ingroup identification were fixed at zero. Also, for the sake of parsimony, only intercepts (and not slopes) for the political variables were regressed on the control variables (i.e., age, sex, and education). Figure 1 conveys other details of the model specification. Note that, given the model specification, the intercepts equal the values of variables at Time 1 (i.e., their initial levels).

This model included several parameters intended to test our research questions (indicated by bolded paths in Figure 1). First, the intercept of TPID, which corresponds to the initial level of this variable, was regressed on the intercepts of each political variable. This directional decision reflects our view that scores for the political variables are temporally antecedent to those for TPID, despite their being measured concurrently at Time 1. Initial levels of the political variables explained 60% of the variation in initial level of TPID. Second, to test whether change in the political variables varied as a function of initial level of TPID, the slope for each political variable was regressed on the intercept for TPID. Finally, to examine whether change in TPID was related to the Time 1 level of any of the political variables, the TPID slope was regressed on the intercept for each variable. This model provided a close fit to the data [χ^2^(222) = 253.01, *p* = .08; CFI = .99; TLI = .99; RMSEA = .02]. The results of the model (summarized in Tables 2 and 3) yielded several key findings.

**Table 2:** Regression Paths, Residual Means, and Residual Variances for the Final Model.

Parameter		B	95% CI				β	
			Lower		Upper			
Regression Paths								
TPID_I_ on	PREJ_I_	−0.11	−0.36	0.15		−0.06		
	RID_I_	−0.07	−0.29	0.16		−0.04		
	LIB_I_	0.33**	0.10	0.55		0.21		
	SOCCON_I_	0.12^+^	−0.02	0.26		0.12		
	SDO_I_	0.21^+^	−0.01	0.43		0.14		
	NATDEC_I_	0.61***	0.40	0.81		0.51		
ΔTPID on	TPID_I_	0.03	−0.03	0.09		0.40		
	PREJ_I_	−0.03	−0.13	0.07		−0.24		
	RID_I_	0.05	−0.04	0.14		0.40		
	LIB_I_	−0.11*	−0.20	−0.02		−1.03		
	SOCCON_I_	0.05^+^	0.00	0.11		0.76		
	SDO_I_	−0.02	−0.10	0.07		−0.18		
	NATDEC_I_	0.00	−0.08	0.08		0.02		
ΔPREJ on	PREJ_I_	0.02	−0.05	0.09		0.35		
	TPID_I_	0.02	−0.01	0.06		0.84		
ΔRID on	RID_I_	0.02	−0.06	0.11		0.26		
	TPID_I_	0.05^*^	0.01	0.09		0.99		
ΔLIB on	LIB_I_	−0.01	−0.06	0.03		−0.14		
	TPID_I_	0.00	−0.02	0.03		0.09		
ΔSOCCON on	SOCCON_I_	−0.01	−0.07	0.05		−0.08		
	TPID_I_	0.00	−0.05	0.06		0.02		
ΔSDO on	SDO_1_	0.02	−0.04	0.08		0.11		
	TPID_1_	0.00	−0.03	0.04		0.02		
ΔNATDEC on	NATDEC_1_	−0.06	−0.14	0.03		−0.36		
	TPID_1_	0.03	−0.04	0.10		0.22		
Residual Means								
	TPID_I_	−1.85***	−2.84	−0.87				
	PREJ_I_	3.02***	2.89	3.15				
	RID_I_	2.69***	2.55	2.83				
	LIB_I_	3.49***	3.35	3.63				
	SOCCON_I_	2.64***	2.42	2.87				
	SDO_I_	2.43***	2.29	2.58				
	NATDEC_I_	3.11***	2.93	3.30				
	ΔTPID	0.09	−0.30	0.49				
	ΔPREJ	−0.07	−0.27	0.12				
	ΔRID	−0.10	−0.34	0.15				
	ΔLIB	0.03	−0.11	0.16				
	ΔSOCCON	−0.01	−0.14	0.13				
	ΔSDO	−0.02	−0.14	0.10				
	ΔNATDEC	0.11	−0.10	0.32				
Residual Variances								
	TPID_I_	0.49***	0.38	0.61				
	PREJ_I_	0.34***	0.27	0.42				
	RID_I_	0.37***	0.27	0.46				
	LIB_I_	0.51***	0.42	0.59				
	SOCCON_I_	1.26***	1.03	1.50				
	SDO_I_	0.51***	0.41	0.61				
	NATDEC_I_	0.80***	0.64	0.96				
	ΔTPID	0.00						
	ΔPREJ	0.00						
	ΔRID	0.00						
	ΔLIB	0.00	−0.01	0.01				
	ΔSOCCON	0.02	−0.01	0.05				
	ΔSDO	0.02^+^	0.00	0.03				
	ΔNATDEC	0.02	0.00	0.05				

*Note.* ^+^ *p*<.10, ^*^ *p*<.05, ^**^ *p*<.01, ^***^ *p*<.001. TPID = Tea Party identification, PREJ = racial prejudice, RID = racial identity, LIB = Libertarianism, SOCCON = social conservatism, SDO = Social Dominance Orientation, NATDEC = belief in national decline. An ‘I’ following the abbreviation indicates a latent intercept. Residual variances marked with ‘*a*’ were constrained to 0 to enable model convergence.

**Table 3:** Covariance Paths in the Final Model.

Parameter		B	95% CI		β
			Lower	Upper	
PREJ_I_ with	RID_I_	0.05^+^	0.00	0.10	0.14
	LIB_I_	0.11***	0.05	0.16	0.26
	SOCCON_I_	0.19***	0.10	0.28	0.29
	SDO_I_	0.25***	0.19	0.31	0.60
	NATDEC_I_	0.21***	0.14	0.29	0.41
RID_I_ with	LIB_I_	−0.14***	−0.20	−0.08	−0.32
	SOCCON_I_	−0.10^+^	−0.19	0.00	−0.14
	SDO_I_	0.03	−0.03	0.09	0.07
	NATDEC_I_	0.04	−0.04	0.11	0.07
LIB_I_ with	SOCCON_I_	0.50***	0.39	0.61	0.63
	SDO_I_	0.21***	0.14	0.27	0.41
	NATDEC_I_	0.36***	0.27	0.45	0.57
SOCCON_I_ with	SDO_I_	0.34***	0.23	0.45	0.42
	NATDEC_I_	0.61***	0.47	0.76	0.61
SDO_I_ with	NATDEC_I_	0.34***	0.25	0.43	0.53

#### Associations between tea party identification and political variables

We first examined relationships between the latent intercepts for Tea Party identification and the political variables. Higher initial levels of libertarianism and belief in national decline predicted higher initial levels TPID while controlling for other parameters in the model and allowing covariances among the political variables. Initial levels of social conservatism and SDO were also positively associated with TPID, but at trend levels (*p*<.10).

Although we observed a bivariate correlation between Time 1 racial prejudice and Tea Party identification (Table 1), we saw no association between the latent intercepts for these constructs after controlling for other variables in the SEM. Seeking to explain this, we tested whether the effect of prejudice on Tea Party identification was mediated by another variable in the model. Indeed, we found that the association between the latent intercepts for prejudice and TPID was significantly mediated by the assertion of national decline (β_indirect_ = .20, *z* = 4.25, *p*<.001) and, to a lesser extent, by libertarianism (β_indirect_ = .06, *z* = 2.29, *p*<.05). In other words, highly prejudiced participants tended to report high levels of national decline and libertarianism, which in turn predicted identification with the Tea Party movement.

#### Change in tea party identification as a function of political variables

Change over time in TPID was associated with the initial levels of two political variables–libertarianism and social conservatism–but in opposite ways. Compared to participants low in libertarian ideology at Time 1, strong libertarians showed steeper declines in TPID. In contrast, participants high in initial social conservatism tended to increase more (or decrease less) in TPID than did socially liberal participants (at a trend level, *p* = .056).

#### Change in political variables as a function of tea party identification

The slope of only one construct–namely, White racial identity–varied as a function of initial levels of TPID. Compared to individuals low in TPID at Time 1, individuals high in initial identification with the Tea Party movement tended to increase more (or decrease less) in White racial identity.

## Discussion

**Figure 2:**
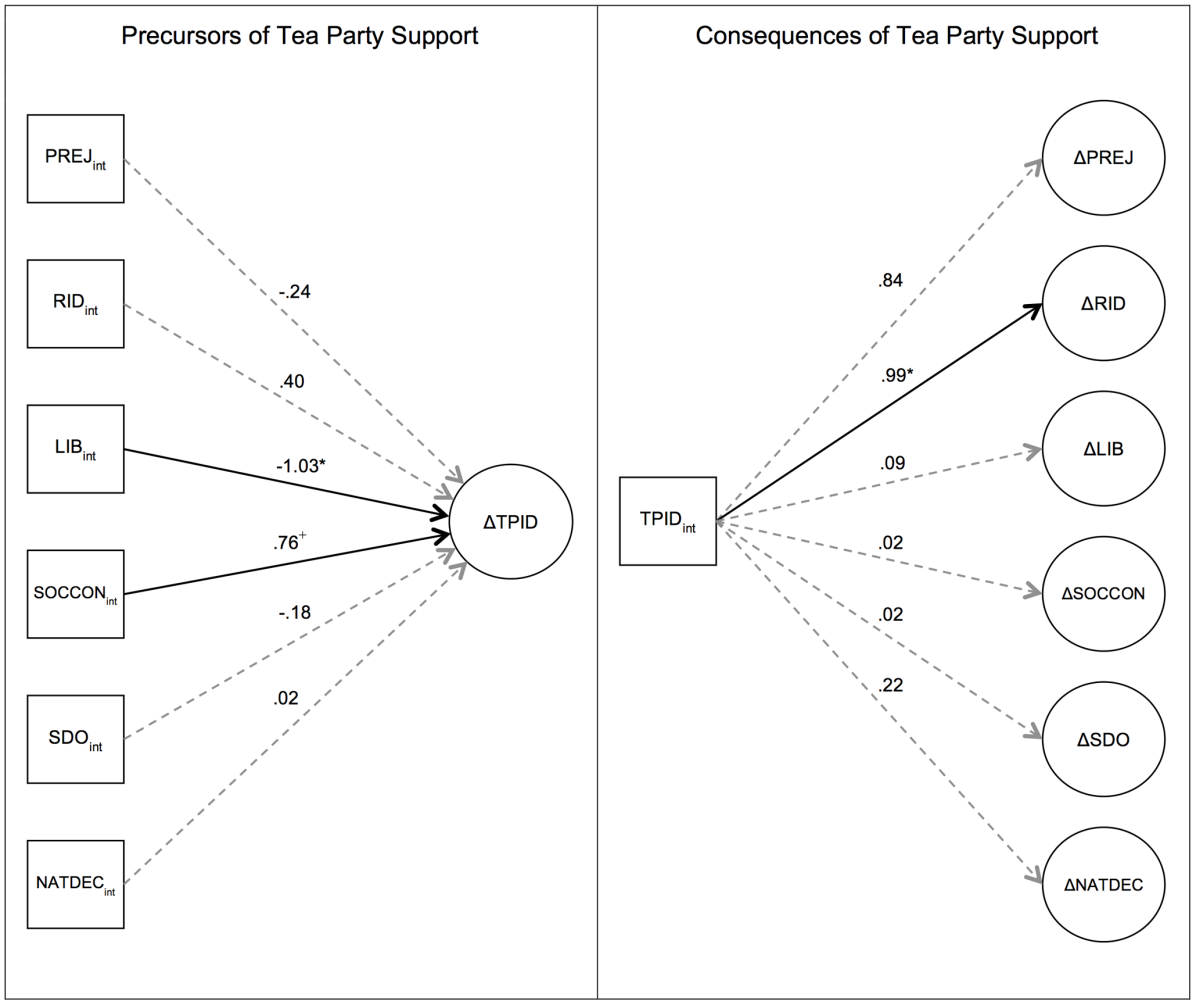
Summary of model results. *Left panel:* Change in Tea Party support as a function of Time 1 values of racial and ideology measures. *Right panel:* Racial and ideology measures as a function of Tea Party identification at Time 1. TPID = Tea Party identification, PREJ = prejudice, RID = racial identification, LIB = libertarian ideology, SOCCON = social conservatism, SDO = Social Dominance Orientation, NATDEC = belief in national decline. Values represent standardized regression estimates. ^+^ *p*<.10, *p<.05, **p<.01.

The present data provide a rich context in which to examine hypotheses derived from various perspectives on race, ideology, and politics. The most relevant findings are summarized in Figure 2.

### Precursors of Identification with the Tea Party

#### Outgroup-focused factors

The outgroup-focused perspective on race and political judgment, prominent examples of which include symbolic and modern racism theories [13]–[15], postulates that Whites’ political views and behaviors are shaped by their attitudes toward racial outgroups–especially African Americans.

Consistent with the outgroup-focused approach, prejudice was associated with Tea Party identification at the Time 1 assessment (see Table 1). This relationship, however, was mediated by a sense of national decline and libertarian ideology, such that prejudice predicted heightened national decline beliefs and libertarianism, and these variables predicted identification with the Tea Party. A sense of national decline–in particular, that the government and American society is diverging from its founding values–is a major theme within the Tea Party movement [45]. Libertarianism, of course, is one of the movement’s stated bases [45].

This pattern of mediation fits broadly with prominent accounts of “modern” prejudice, including symbolic racism theory [13] and legitimation theories [62], which suggest that “old-fashioned” racism has faded as a political force in the United States. People are aware of the cultural opprobrium that attaches to undisguised antipathy. Thus, racial resentment becomes couched in terms of more symbolic, philosophical, complaints about Black culture–for instance, that Blacks tend to lack industry and seek undeserved government assistance, as well as legitimizing beliefs that do not refer directly to Blacks at all. Symbolic racism theory stresses the former process, in which Whites come to view Blacks as flouting “traditional” American values of individualism and self-reliance. Legitimation theories, such as social dominance theory [59] and system justification theory [67], cast these group-neutral values in a somewhat different role–not as bases for a complaint about Blacks per se, but as principles that justify particular political preferences (e.g., opposition to affirmative action, support for “small government,” etc.). The present results are consistent with the notion that race-based support for the Tea Party is legitimized by the assertion of value-based cultural complaints and libertarian/individualist ideology [61].

#### Ingroup-focused factors

Ingroup-focused approaches to race and political judgment, including realistic group conflict [21], group position [22], [23], and certain social identity approaches [24], posit that Whites’ political judgments are driven, in part, by their concern for the material prospects or social status of their racial ingroup. Ingroup identification ought, under this approach, to serve as a proxy for individuals’ degree of ingroup-focused concern. However, the data offered no evidence that racial identity causes identification with the Tea Party. Within the Time 1 assessment, racial identity was negatively correlated (albeit weakly) with Tea Party identification (Table 1). In the latent growth model, which controls for the other political variables, Time 1 levels of racial identity were not associated with Tea Party identification. Moreover, we did not find that racial identity at Time 1 predicted change in Tea Party identification over the course of study.

#### Ideological factors

The ideological approach to political judgment rejects the role of prejudice and racial identity in Whites’ political attitudes and behavior. Instead, political judgment is seen as reflecting genuine ideological commitments, such as political conservatism [12], [68], [69]. Our data point to a connection between small-government (i.e., libertarian) ideology–the stated inspiration for the Tea Party–and identification with the movement: At Time 1, libertarianism covaried significantly and independently with Tea Party support. However, between July 2010 and May 2011, it appears that strong libertarians became progressively disenchanted with the Tea Party movement, with high levels of libertarian ideology at Time 1 associated with steeper declines in Tea Party identification over the following nine months.

Unlike libertarianism, social conservatism appeared to drive identification with the Tea Party over the course of the study. Not only was social conservatism independently associated with Tea Party identification at Time 1, but socially conservative Whites tended to increase more (or decrease less) in their support for the Tea Party over the subsequent nine months. These data support the idea that the Republican Party’s attempts to harness the Tea Party have diluted its original emphasis on economic issues, rendering the movement less appealing to libertarians and more appealing to social and cultural conservatives [46]–[48].

### Evidence for Political Racialization

Our data support the notion that political affiliations can lead to systematic changes in individuals’ racial thinking. Strong identification with the Tea Party at Time 1 predicted longitudinal increases in White racial identity. Thus, alignment with the Tea Party appears to have increased Whites’ consciousness of membership in the dominant racial group–a pattern that closely parallels previous findings in which membership in racially homogeneous organizations increased Whites’ levels of racial identity [41]. Thus, the results provide initial support for the hypothesized process of political racialization, and suggest that future research continue to examine this mechanism by which individuals’ racial concerns and political judgments become aligned.

### The Focus on Anti-Black Prejudice

The present study was limited in its focus on the role of anti-Black prejudice in the Tea Party. We had several reasons for this. First, the Tea Party is a movement inspired in part by opposition to Barack Obama, the United States’ first Black president. Second, anti-Black prejudice has already been shown to predict political attitudes, such as opposition to heath care reform, that play a salient role in Tea Party rhetoric [6], [7]. Third, in American culture, the Black group has long been the primary target of Whites’ racial resentments [70], although this may be expanding to include other groups. Finally, there are theoretical reasons to believe that individual group prejudices reflect an underlying, general prejudicial tendency, and that similar results would emerge if our measure of anti-Black prejudice were replaced with (e.g.) anti-immigrant bias [71]. Nonetheless, and especially given anti-immigrant sentiment among many Tea Party supporters, future research should expand the analysis to include attitudes toward immigrants and other stigmatized social groups.

### Conclusion

The present paper examined possible causes and consequences of identification with the Tea Party movement. We measured longitudinal change in Tea Party identification and an array of constructs relevant to three approaches to the relationship between racial thinking and political judgment–namely, the *outgroup-focused*, *ingroup-focused*, and *ideological* perspectives.

Broadly, the data support claims that the Tea Party is–for some White supporters, at least–a racially motivated movement. Anti-Black sentiment was associated with Tea Party identification across time points. This relationship, however, appeared to be masked by assertions of national decline and the embrace of libertarian ideology.

The findings also suggest that identification with the Tea Party movement is related to racial identity, but not in the manner suggested by traditional models of racialized politics. Rather than causing affiliation with the Tea Party, White identity appears to be a product of immersion in the movement [41]. This phenomenon, which we term *political racialization*, merits further study to reveal the precise mechanisms by which identification with a political movement can shape racial attitudes and identities.

Our findings concerning libertarianism and social conservatism shed light on the ideological dynamics of the Tea Party movement during an important time in its history. Although support for the Tea Party movement tended to fall over the study period–from June 2010 to April-May 2011–the movement retained greater appeal for social conservatives than for libertarians. Thus, it may be that Republican attempts to exploit enthusiasm for the Tea Party succeeded in shifting the movement (in popular perception, at least) from economic to culturally conservative themes.

## Supporting Information

Appendix S1 (DOCX)
