# Supplementary material for: Race, Ideology, and the Tea Party: A Longitudinal Study
Source: PLoS One. 2013 Jun 25;8(6):e67110. doi: 10.1371/journal.pone.0067110 (PMC3692430; doi:10.1371/journal.pone.0067110)
Supplement: Appendix S1 — (DOCX) [file pone.0067110.s001.docx]

**Appendix S1**

Anti-Black scale [1] item subset

1. Most Blacks have the drive and determination to get ahead (reversed).
2. The root cause of most of the social and economic ills of Blacks is the weakness and instability of the Black family.
3. On the whole, Black people don’t stress education and training.
4. Black children would do better in school if their parents had better attitudes about learning.
5. One of the biggest problems for a lot of Blacks is their lack of self-respect.

**Racial Common Fate scale [2] item subset**

1. What happens to my racial group as a whole in this country will have something to do with what happens in my life.
2. My opportunities in life are tied to those of my racial group as a whole.
3. My fortunes in life can be expected to rise and fall with those of my racial group as a whole.
4. Because of my unique experiences and personal qualities, my opportunities aren’t influenced by the changing fortunes of my racial group as a whole (reversed).

**Libertarianism-Totalitarianism scale [3]**

1. Excessive taxation is a prime example of the way in which governments take away individual freedom.
2. We need a stronger government to create a better society (reversed).
3. Government programs discourage individual responsibility and achievement while fostering dependency and failure.
4. In my kind of ideal society, all basic needs (food, housing, healthcare, education) will be guaranteed by the government for everyone (reversed).
5. The more powerful a government becomes, the greater is the risk that it will become corrupt and unresponsive to the will of its people.
6. A fair society is not possible without strict and comprehensive government controls (reversed).
7. Individuals create wealth and governments tax it away to promote the interests of those in control.
8. Individual freedom and opportunity are greater when government is smaller and less able to intervene in social and economic areas.
9. Government laws and regulations make it possible to have a moral society (reversed).
10. I am entitled only to the fruits of my own labor; not to that of others passed on to me through government handouts.
11. I am willing to exchange my personal freedoms for greater security provided by government programs (reversed).
12. We need strict government intervention to ensure that everyone will succeed socially and economically (reversed).
13. Typically, government agencies spend our money carelessly and wastefully, which is natural, since they don’t have to earn it.
14. Most of our economic woes are caused by repeated and massive government meddling in the economy.
15. Our government is not active enough; we need more laws and government programs to regulate and improve our lives and dealings with each other (reversed).
16. As a government gets bigger and more powerful, its citizens become poorer and less free.
17. For me, government-imposed social order and security are more important than individual freedom (reversed).
18. Our society can improve only with more government controls over individuals and businesses (reversed).
19. My ideal government would be very small and would only perform a very few essential functions.
20. Government must limit our individual freedoms so as to prevent unchecked selfishness, greed, and immorality (reversed).

**Social Dominance Orientation scale [4] item subset**

1. If certain groups stayed in their place we would have fewer problems.
2. It’s probably a good thing that certain groups are at the top and other groups are at the bottom.
3. Inferior groups should stay in their place.
4. We should do what we can to equalize conditions for different groups (reversed).
5. Group equality should be our ideal (reversed).
6. We would have fewer problems if we treated different groups more equally (reversed).

**References**

1. Katz I, Hass RG (1988) Racial ambivalence and American value conflict: Correlational and priming studies of dual cognitive structures. J Pers Soc Psychol 55: 893-905.
2. Lowery BS, Knowles ED, Unzueta MM (2007) Framing inequity safely: Whites’ motivated perceptions of racial privilege. Pers Soc Psychol Bull 33: 1237-1250.
3. Mehrabian A (1996) Relations among political attitudes, personality, and psychopathology assessed with new measures of libertarianism and conservatism. Basic Appl Soc Psych 18: 469-491.
4. Pratto F, Sidanius J, Stallworth LM, Malle BF (1994) Social dominance orientation: A personality variable predicting social and political attitudes. J Pers Soc Psychol 67: 741-763.
